# Host immunity in the protective response to nasal immunization with a pneumococcal antigen associated to live and heat-killed *Lactobacillus casei*

**DOI:** 10.1186/1471-2172-12-46

**Published:** 2011-08-11

**Authors:** Elisa O Vintiñi, Marcela S Medina

**Affiliations:** 1 Laboratorio de Bioquímica y Clínica Experimental. Centro de Referencia para Lactobacilos (CERELA-CONICET). Chacabuco 145. Tucumán. Argentina; 2 Facultad de Agronomía y Zootecnia. Florentino Ameghino S/N. El Manantial. Universidad Nacional de Tucumán. Tucumán. Argentina; 3 Facultad de Bioquímica, Química y Farmacia. Ayacucho 495. CP: 4000. Universidad Nacional de Tucumán. San Miguel de Tucumán. Tucumán. Argentina

## Abstract

**Background:**

At present, available pneumococcal vaccines have failed to eradicate infections caused by *S. pneumoniae*. Search for effective vaccine continues and some serotype independent pneumococcal proteins are considered as candidates for the design of new vaccines, especially a mucosal vaccine, since pneumococci enter the body through mucosal surfaces. Selection of the appropriate adjuvant is important for mucosal vaccines, and lactic acid bacteria (LAB) with immunostimulant properties are promissory candidates. In this work, we assessed the adjuvant effect of a probiotic strain, Lactobacillus casei (*L. casei*), when nasally administered with a pneumococcal antigen (pneumococcal protective protein A: PppA) for the prevention of pneumococcal infection. Adjuvanticity of both live (LcV) and heat-killed (LcM) was evaluated and humoral and cellular antigen-specific immune response was assessed in mucosal and systemic compartments. The potential mechanisms induced by nasal immunization were discussed.

**Results:**

Nasal immunization of young mice with PppA+LcV and PppA+LcM induced anti-PppA IgA and IgG antibodies in mucosal and systemic compartments and levels of these specific antibodies remained high even at day 45 after the 3rd Immunization (3rd I). These results were correlated with IL-4 induction by the mixture of antigen plus LcV and LcM. Also, PppA+Lc (V and M) induced stimulation of Th1 and Th17 cells involved in the defence against pneumococci. The protection against pneumococcal respiratory challenge at day 30 after the 3rd I showed that PppA+LcV and PppA+LcM immunizations significantly reduced pathogen counts in nasal lavages while prventing their passage into lung and blood. Survival of mice immunized with the co-application of PppA plus LcV and LcM was significantly higher than in mice immunized with PppA alone and control mice when intraperitoneal challenge was performed. No significant differences between the treatments involving LcV and LcM were found.

**Conclusions:**

Live and heat-killed L. casei enhanced the antigen-specific immune response when administered nasally with a pneumococcal antigen. Considering the potential risk associated with live bacteria, the design of a nasal vaccine based on pneumococcal antigens and heat-killed L. casei emerges as a safe and effective strategy for the prevention of pneumococcal infections and opens new possibilities of application of dead LAB as adjuvants in vaccine formulations against other pathogens.

## Background

Pneumococcal infections are one the most common diseases in both developed and developing countries [1,2]. Despite the widespread use of antibiotics, the emergence of antibiotic resistant *Streptococcus pneumoniae *strains and some problems associated with vaccines have made pneumococcal diseases a major health problem worldwide. At present, there are two types of pneumococcal vaccines in use: capsular polysaccharide pneumococcal vaccines (PPV) and protein-polysaccharide conjugate pneumococcal vaccines (PCV). PPV are not effective in elderly people, immunocompromised individuals or children under 2 years of age, while PCV are expensive for application in developing countries as global vaccination strategies. In addition, recent researches have shown that serotypes eradicated by PCV are being replaced by non-vaccine pneumococcal serotypes [3-5]. Thus, new strategies for the fight against pneumococci are necessary, and pneumococcal proteins conserved among various serotypes represent novel alternatives to develop protein-based pneumococcal vaccines (PBPV). In this sense, some proteins have been identified and their capacity to afford protection against invasive and respiratory infections is being studied [6,7] but at present no protein-based pneumococcal vaccines (PBPV) have been licensed. The use of adjuvants is necessary to reach an adequate mucosal protective immune response and the selection of safe and effective adjuvants is a crucial point in the design of mucosal vaccines. At present, the best studied mucosal adjuvants are Cholera toxin (CT) and *E. coli *lymphotoxin (LT), which are highly immunogenic as well as the most effective experimental adjuvants known today. However, they are also very toxic and not acceptable for human use [8]. In this sense, and based on the "intrinsic" immunostimulator properties and GRAS (Generally recognized as safe) status of certain lactic acid bacteria (LAB) strains [9], some researchers have considered using them as carriers of different pneumococcal antigens to prevent pneumococcal infections. Previous studies demonstrated that immunization with some pneumococcal proteins (e.g. PspA, PsaA, PpmA, PspC) expressed in lactococcus or lactobacillus strains are able to afford protection against *S. pneumoniae *in mouse models [10-14]. In a previous report, we showed that a recombinant lactococcus expressing the pneumococcal protective protein A (PppA), a protein antigenically conserved among different serotype strains of *S. pneumoniae *(3, 5, 9, 14, 19 and 23) [15], was able to afford protection against pneumococcal infection in a mouse model [16,17]. A recent work showed that nasal administration of recombinant *Lactobacillus casei *expressing PspC was able to reduce nasal colonization by *S. pneumoniae *in a mouse model [11]. However, there are no investigations concerning the effect on the immune system of a non-recombinant lactobacillus associated to pneumococcal antigen. The potential application of a recombinant strain in humans still presents aspects that need to be resolved such as elimination of antibiotic resistance genes, production of recombinant strains in a controlled system, safety of its application to children and immunosuppressed individuals and cost-benefit evaluation of its production, especially in developing countries. In the last few years, the use of some LAB strains in the prevention of respiratory infection has become more evident [18-21]. In this sense, the oral and nasal administration of live *Lactobacillus casei *CRL 431 (*L. casei*), a probiotic strain, was able to improve the immune response of the host against respiratory pathogens in a mouse model. The effect of *L. casei *was associated with the stimulation of the innate and adaptative immune response [18,20-22]. In addition, some studies have demonstrated that non-viable lactobacilli also have immunostimulant properties in both humans and animals [23-25], so their use in nasal vaccines is an interesting point to asses. At present, only a few articles have assessed the immune effect of the administration of non- recombinant lactic acid bacteria associated to a specific antigen [26] and investigations about a pneumococcal antigen associated to heat-killed LAB have not been reported. Most publications evaluate recombinant lactic acid bacteria expressing pneumococcal Ag as potential vaccines against *S. pneumoniae *[10-14]. In contrast, in this work a new option, based on non-engineered and non viable lactic acid bacteria, was explored as a potential strategy for nasal vaccination against *S. pneumoniae*.

On the other hand, since *S. pneumoniae *enters the host primarily through the respiratory mucosa, vaccination strategies by the nasal route are of great interest, especially if we take into account the fact that most vaccines delivered parenterally are not completely effective to induce mucosal immunity [27]. Moreover, the nasal route proved to be the most effective pathway to induce protective immunity against the pneumococcus [28-31]. Since the use of probiotics implies an adequate selection of strains for specific applications [32], we hypothesized that a combination of pneumococcal antigen with *Lactobacillus casei *as a nasal adjuvant could be able to trigger protective immune responses against pneumococcal infection. On the basis of the above considerations, the aim of this work was to assess the mucosal and systemic immune response induced by nasal immunization with a pneumococcal antigen (PppA) associated to live and heat-killed *Lactobacillus casei *and to analyze the possible immune mechanisms involved in its protective effect.

## Results

### Enhancement of protective systemic and mucosal antibody response by inclusion of live and heat-killed L. casei in nasal vaccination with PppA

**Figure 1:**
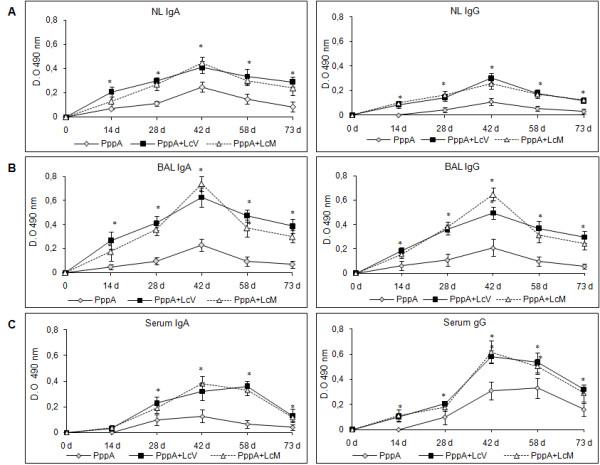
**Immunoglobulin (Ig)A (a) and IgG (b) anti-pneumococcal protective protein A (PppA) antibodies response in nasal and bronchoalveolar lavages (NL and BAL) and serum of young mice nasally immunized with PppA and PppA associated with live (LcV) and heat-killed (LcM) *Lactobacillus casei *CRL 431**. Results are expressed as the mean of the optical density (OD) ± standard deviation for each specific Ig (*n *= 6 mice/group at each time point). Antibodies were determined using the enzyme-linked immunosorbent assay method and samples were considered negative for the presence of specific antibodies when OD 493 < 0.1. Results are representative of two independent experiments. Significant differences with the PppA group were: *P *< 0.01.

Mucosal IgA and IgG antibodies are important for the defence against respiratory infections because, while IgA prevents pathogen attachment to epithelial cells, thus reducing colonization, IgG promotes phagocytosis and prevents local dissemination of the pathogen in the alveoli as well as its passage into blood [33]. At the systemic level, IgG antibodies ensure protection against invasive disease. To evaluate the effect of PppA, PppA+LcV and PppA+LcM on specific antibody responses in the mucosal and systemic compartment, groups of six mice for each time were immunized i.n. with PppA, PppA+LcV, PppA+LcM, LcV, LcM and PBS, according to protocols previously described. Samples of NL, BAL and serum analyzed on days 0, 14, 28, 42, 58 and 73 showed that nasal immunization of young mice with PppA, PppA+LcV and PppA+LcM induced specific anti-PppA IgA and IgG antibodies in three types of samples, while no detectable values of these specific antibodies were observed in any of the samples collected from animals that received LcV, LcM or PBS (Figure 1). In NL, the levels of IgA and IgG anti-PppA induced by PppA+LcV and PppA+LcM were very similar and higher than antibodies levels induced by PppA group, while in BAL the results were different. Thus, PppA+LcM tended to higher IgA values than PppA+LcV on day 42, but no significant differences were obtained. In addition, PppA+LcM group induced higher specific IgG levels than PppA+LcV in BAL on day 42 and significantly differences were found. In serum, both PppA+LcV and PppA+LcM treatments induced similar IgA and IgG anti-PppA levels. Overall, animals that received PppA alone produced very low mucosal and serum anti-PppA responses and antibody levels, reaching a small peak after the third immunization (day 42) and then decreasing considerably. Thus, immunization with PppA failed to induce sustained levels of specific antibodies over time. In contrast, the nasal administration of PppA+LcV and PppA+LcM significantly increased IgA and IgG antibody levels compared with PppA and its levels remained high even a month and a half after the third immunization. Our results suggest that both LcV and LcM were able to enhance the specific humoral immune response at the mucosal and systemic levels when they were associated with a pneumococcal antigen.

### Pattern of cytokines induced by nasal immunization

**Figure 2:**
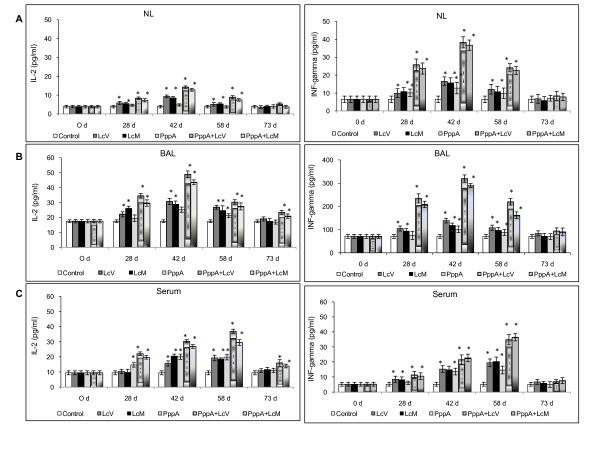
**T helper type 1 (Th1) cytokines production in Nasal lavages = NL (A), Bronchoalveolar lavages = BAL (B) and Serum (C) of young mice stimulated with protective pneumococcal protein A (PppA) and PppA associated with *Lactobacillus casei *(PppA+LcV and PppA+LcM)**. Nasal administration of *L. casei *was assessed. Non-stimulated young mice that received PBS were also evaluated as controls of basal cytokine levels (control). Results are expressed as mean ± standard deviation (*n *= 6 mice/group at each time point). Significantly different from the control group: *P < 0.01.

In order to evaluate the cellular T immune response stimulated by nasal vaccination, Th1, Th2 and Th17 cytokines profiles were studied in mucosal and systemic compartments. In mucosal fluids (NL and BAL), nasal immunization with PppA+LcV and PppA+LcM increased IL-2 and INF-γ levels (days 28, 42 and 58) compared with basal values. PppA+LcV induced higher IL-2 levels than PppA+LcM on days 73 in NL and on days 28 and 42 in BAL, while INF-γ levels on day 58 in BAL also were higher for PppA+LcV compared to LcM+PppA (*p *< 0.05). LcV, LcM and PppA alone showed a variable pattern of Th1 cytokine induction throughout the period assessed (Figure 2A, B and 2C); however, cytokine levels induced by these three groups were lower than PppA+LcV and PppA+LcM. Both LcV and LcM were able to induce Th1 cytokines in mucosal compartments. In serum, PppA+LcV and PppA+LcM immunizations increased IL-2 levels during all periods evaluated, while INF-γ was increased on days 28, 42 and 58 in both groups. PppA+LcV showed significant differences with PppA+LcM on day 58 for IL-2 (p < 0.05) cytokine induction while no differences were found for INF-γ production between the two groups. Immunization with PppA alone was not very effective to stimulate INF-γ and this cytokine increased only on day 42 in mucosal fluids, while in serum it increased only on days 42 and 58. Live (LcV) and heat-killed (LcM) *Lactobacillus casei *increased Th1 IL-2 and INF-γ cytokines on days 28, 42 and 58 in mucosal fluids while in serum only INF-γ was increased on those three days; however, these cytokine levels were lower than those induced in the groups that included *L. casei *associated with the pneumococcal antigen.

**Figure 3:**
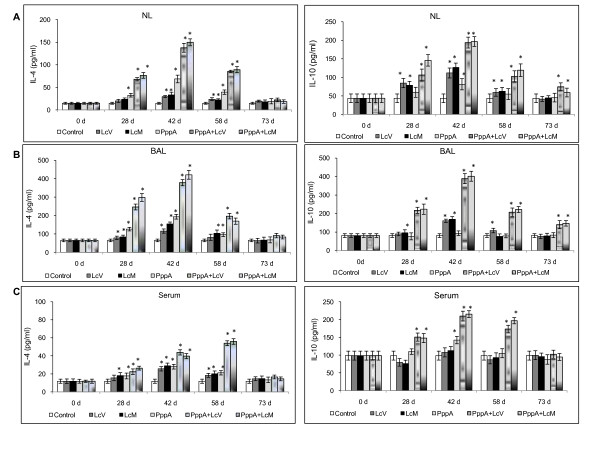
**Mucosal (nasal = NL (A) and bronchoalveolar lavages = BAL (B)) and systemic (serum (C)) T helper type 2 (Th2) cytokines production by young mice immunized with different groups of PppA, *Lactobacillus casei *(Lc) and PppA+Lc vaccines**. (see Fig. 2 for details).

**Figure 4:**
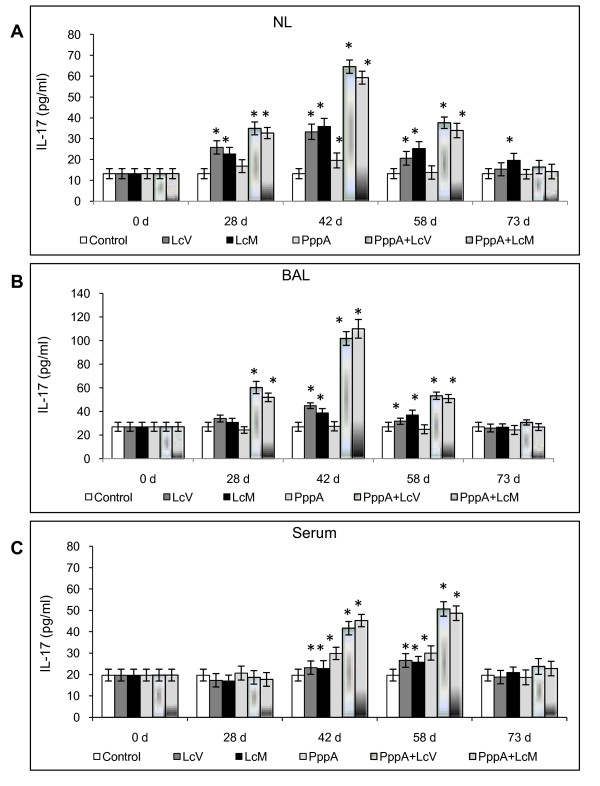
**Serum, Nasal and Bronchoalveolar lavages (NL and BAL) IL-17A production by young mice immunized with different treatments containing PppA and Lc**. (see Fig.2 for details).

On the other hand, during almost all periods evaluated, nasal immunization with the different treatments induced high levels of IL-4 in both mucosal and systemic compartments compared with basal levels (Figure 3A, B and 3C). However, the highest level of this cytokine, which is a marker of the stimulation of Th2 cells, was obtained with the nasal administration of live and dead *L. casei *associated with the pneumococcal protein PppA. These results are consistent with the specific humoral response induced in the PppA alone, PppA+LcV and PppA+LcM groups. In addition, PppA+LcM induced higher IL-4 values than PppA+LcV (*P *< 0.05) on day 28 in BAL, while no significant differences in this cytokine were observed for other periods in mucosal fluids neither in serum. The regulatory IL-10 cytokine showed a variable behaviour, depending on the experimental group studied. However, PppA+LcV and PppA+LcM induced higher values of IL-10 in both mucosal and systemic compartments during almost all periods evaluated. This fact would be important to ensure a balanced immune response against a pneumococcal infection. Finally, IL-17A, which represents activation of the Th17 cells, was also evaluated (Figure 4A, B and 4C). This cytokine showed a variable pattern depending on experimental group and post-immunization days considered. In NL and BAL, nasal administration of LcV, LcM induced an increase of IL-17 levels with respect to control group, while that LcM also increase this cytokine on day 73, in NL. In addition, IL-17 was increased in PppA+LcV and PppA+LcM groups at days 28, 42 and 58 in both NL and BAL. In contrast, PppA just induced an increase in IL-17 in NL at day 42. In serum, the LcV, LcM, PppA, PppA+LcV and PppA+LcM groups induced a significant increase of IL-17 on days 42 and 58 compared with the control group. Considering that IL-17 is involved in the defence against *S. pneumoniae*, these results are very important. Overall, the evaluation of different cytokines associated with Th cells populations showed that both live and heat-killed *L. casei *(LcM) was able to exercise an important adjuvant effect on the specific cellular and humoral immune response. Thus, a good antigen-specific immune response was reached with the mixtures: pneumococcal Ag+LcV and pneumococcal Ag+LcM.

### Protection against respiratory tract infection after nasal pneumococcal challenge

A pneumococcal respiratory infection model was used to assess nasal and lung colonization as well as dissemination of the pathogen into blood. Nasal immunization with the pneumococcal antigen associated with live (LcV) and heat-killed (LcM) *L. casei *was able to prevent pneumococcal colonization in lung and pathogen dissemination in blood, showing negative hemocultures (Table 1). Also, co-administration of PppA with LcV and LcM significantly reduced pathogen counts in nasal lavages compared with PppA and control groups. Also, an important hallmark was that both live and heat-killed *L. casei *were able to reduce mucosal colonization and prevent the passage of pneumococci into blood, without significant differences between them. However, pathogen counts in mucosa compartments were higher than the ones found after antigen specific + *L. casei *(LcV and LcM) immunization. PppA+LcV and PppA+LcM immunizations were able to reduce pathogen counts in NL significantly and prevented their passage into lung and blood. Finally, immunization with PppA alone induced a lower reduction in the number of *S. pneumoniae *in NL and lung compared with the other experimental groups, except for the control. In addition, this antigen alone did not prevent pathogen passage into blood.

**Table 1:** Nasal, lung and blood colonization by *S. pneumoniae *after respiratory challenge with the pathogen

Groups	*S. pneumoniae *serotype 14		
	**NL**	**Lung**	**Blood**

**Control**	5.67 ± 0.23^a^	6.79 ± 0.12 ^a'^	4.42 ± 0.21^a''^

**PppA**	4.98 ± 0.32^b^	5.53 ± 0.51^b'^	3.92 ± 0.14^b''^

**LcV**	4.10 ± 0.18^c^	3.16 ± 0.14^c'^	<1.5^c''^

**LcM**	4.26 ± 0.22^c^	3.28 ± 0,17^c'^	<1.5^c''^

**PppA+LcV**	2.38 ± 0.21^e^	<1.5^e^	<1.5^c''^

**PppA+LcM**	2.53 ± 0.19^e^	<1.5^e^	<1.5^c''^

Mice were immunized with treatments containing PppA, LcV and LcM. Animals receiving phosphate-buffered saline (PBS) were used as controls. Results are expressed as log colony-forming units (CFU)/g of lung or log CFU/ml of nasal lavages (NL) and blood. The lower limits of bacterial detection were 1.5 log CFU/g of lung and 1.5 log CFU/ml of NL and blood, respectively. Significant differences among groups were established by using the least significant difference test (LSD). Means in the table with different letters (a-f, a'-b' or a"-b") were significantly different *P *< 0.05).

### Enhanced resistance to systemic pneumococcal challenge in immunized mice

In order to determine whether the induction of specific humoral and cellular immune response induced by nasal immunization with PppA+LcV and PppA+LcM was correlated with increased resistance to pneumococcal infection, young mice were intraperitoneally challenged with *S. pneumoniae *serotype 14 four weeks after the third immunization.

Animals that received the PppA protein alone or PBS were also evaluated. In both systemic and respiratory challenge assays, *S. pneumoniae *serotype 14 was used because of its high potential for invasive disease and also because it is the prevalent one in our country.

**Figure 5:**
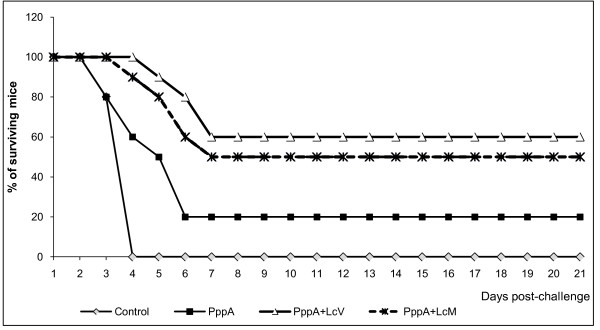
**Survival of young mice intraperitoneally challenged with 10^8 ^cells of *S. pneumoniae *serotype 14 nasally immunized with PppA and PppA+LcV and PppA+LcM**. Mice nasally given PBS were used as control (14 mice/group).

Young mice immunized with PppA+LcV showed a survival percentage of 60% on day 21 after challenge (Figure 5), which was significantly higher compared with the control group that received PBS (*p *< 0.01 Cox's Mantel Test), in which 100% of the mice died after day 4 post challenge. PppA+LcM showed a survival percentage of 50% with respect to PBS group but no significant differences were found compared with PppA+LcV. Mice that received PppA alone showed a survival percentage of 20%, with no significant differences with control. The animals in this group died between days 4 and 6 post challenge. These results demonstrate that both LcV and LcM are effective as adjuvants when they are associated with a pneumococcal antigen because PppA+LcV as well as PppA+LcM immunizations increased resistance to the systemic challenge with *S. pneumoniae *serotype 14.

## Discussion

The licensed pneumoccocal vaccines (PPV, PCV), formulated as vaccines depending on serotypes, did not enable the eradication of infections caused by *S. pneumoniae*. A new conjugate vaccine PCV13 includes 6 additional serotypes with respect PCV7 and provides a broader spectrum of protection than the vaccines mentioned above [34]. However, the emergence of new serotypes as prevalent causes of disease requires the development of new conjugate vaccines with broader serotype coverage. In addition, the high cost of conjugate vaccines and the prevalence of specific pneumococcal serotypes in different parts of the world are obstacles that should be taken into consideration and vaccines should tailored to each geographical region to ensure the greatest level of protection. In view of the above, the design of vaccines that can afford serotype-independent protection emerges as a valuable alternative to control pneumococcal infection around the world. On the other hand, although the pneumococcus enters the body through the respiratory tract, all available vaccines are applied by the parenteral pathway while the mucosal route would be more convenient. Nasal immunization against respiratory pathogens has certain advantages over other pathways of antigen delivery, principally because it mimics the route of entry of pathogens and induces a protective immune response in both the local and systemic compartments. In addition, the cost-benefit analysis as well as their easy and convenient application contributes to the selection of nasal vaccines for immunization against respiratory pathogens. A point to be considered in mucosal vaccines is the selection of appropriate mucosal adjuvants because most vaccine antigens have little or no inherent immunostimulatory properties. At present, very few adjuvants have been approved for clinical use [35], which could be partly due to the cost of development and to the regulatory restrictions imposed on vaccine adjuvants [36] to ensure their effectiveness and safe application to humans. In this sense, LAB are attractive candidates as potential adjuvants, since they are generally recognized as safe and some of them have been proved to have intrinsic adjuvant properties when administered through the mucosal route to prevent respiratory infections [18,20-22]. Our purpose was to assess the adjuvant effect of a specific live and dead probiotic strain when it is associated with a pneumococcal antigenic protein. Thus, we studied the protective effect exerted against *S. pneumoniae *infection by nasal immunization with PppA when live (LcV) and heat-killed (LcM) *Lactobacillus casei *431 was used as a mucosal adjuvant. The role of PppA in the pathogenesis of pneumococcal infection is unknown but it presents homology with a nonheme iron-containing ferritin protein from *Listeria innocua *and other bactoferritins.

PppA was able to induce protective antibodies when it was administered by the nasal route associated with a mucosal adjuvant [15] or when it was expressed in a lactococcus strain and the recombinant bacterium was administered by the oral and nasal routes [17,37,38] in a pneumococcal infection model. When a recombinant strain was used, the intrinsic adjuvant capacity of *Lactococcus lactis *[19,39] enhanced the specific immune response. Although the protection reached in all the above cases was good, the use of recombinant strains have some implications that should be considered such as amount of protein expressed, elimination of ATB markers in live strains, lower adjuvanticity of inactivated recombinant strains that make necessary the implementation of better immunization protocols [37], the design of adequate protocols for human trials and the cost of large-scale production of recombinant strains. In addition, a mixture of proteins may be necessary to achieve a broad-spectrum vaccine against pneumococcal diseases and obtainment of a recombinant strain that expresses various proteins could be difficult. All these aspects should be taken into account for the development of a new vaccine and the use of harmless bacteria as adjuvants emerges as an attractive option. The mixture of different antigens, the control of the concentration of each in vaccine formulations and the selection of strains for particular purposes would be some of the advantages when non-recombinant microorganisms are used. Moreover, non-engineered microorganisms have greater acceptability by the general population than genetically modified ones. Previous reports showed that nasally administered specific lactobacillus and lactococcus strains were able to improve the protective immune response against *S. pneumoniae *[18,19,40,41]. Thus, Cangemi de Gutierrez et al. [18] demonstrated that the intranasal administration of *L. fermentum *isolates from the pharynx of BALB/c mice was able to activate macrophages in the respiratory tract and reduced nasal and lung colonization of *S. pneumoniae*. Also, in previous investigations, we demonstrated that the nasal administration of *Lactococcus lactis (*LL*) *enhances the protective immune response against a pneumococcal challenge in a mouse model [19] and this effect was associated with the up-regulation of the innate and adaptative immunity in both the respiratory and the systemic compartments. In all these studies, live bacteria were used. In contrast, Hori et al. [41] reported that nasal administration of killed-heat *L. casei *Shirota activated the cellular immune response in the respiratory tract and protected against Influenza Virus infection. Recently, it was reported that both live and dead *Lactobacillus casei *431, the ones used in this study, improved protection against pneumococcal infection in malnourished mice [42]. All these reports studied the immunostimulant properties of specific strains of lactic acid bacteria alone. In this work, we demonstrated the adjuvant effect of live and dead *L. casei *nasally administered when associated with a pneumococcal antigen. In this sense, considering that the humoral immune response is extremely important in the defence against *S. pneumoniae*, specific anti-PppA antibodies were evaluated. Thus, nasal immunization of PppA alone induced low levels of specific IgA and IgG antibodies while the PppA+LcV and PppA+LcM mixtures induced the highest values of antigen-specific antibodies. In a previous report it was demonstrated that passive immunization of naïve mice with serum from animals immunized with recombinant *Lactococcus lactis*-PppA increased the survival percentage against a pneumococcal challenge [17] and these results showed that anti-PppA antibodies are important components of the protective immune response. Anti-PppA IgA would contribute to a decrease in the colonization of the respiratory tract by limiting pathogen attachment to the respiratory epithelium while IgG would have an important role as opsonin in the phagocytosis of organisms [33]. Thus, both types of specific inmunoglobulin are desirable in vaccination-induced protective immunity. At the systemic level, the induction of specific IgG was higher than IgA, as expected, which is important to prevent the systemic dissemination of *S. pneumoniae*. Moreover, it was demonstrated that nasal administration of *L. casei *live and killed heat increased the anti-pneumococcal IgA and IgG antibodies in the local and systemic compartments after a pneumococcal respiratory infection [40]. Also, *L. casei *increased the number and activation of alveolar macrophages and neutrophils in BAL. The phagocytosis process involved in the primary depuration of pneumococci would be facilitated by the specific antibodies. Overall, the immunostimulatory effect of the probiotic strain contributes to the strengthening of the protective immune response after vaccination. On the other hand, the nasopharynx-associated lymphoid tissue (NALT) contains all the immune cells required for the induction and regulation of the mucosal immune response to antigens delivered into the nasal cavity [42]. Hussell and Humphreys [43] suggested that the NALT could fulfil an important role by reducing the pathogen burden to a level that only induces minimal inflammation in the lower lung. In consequence, the intranasal priming of NALT with *L. casei *would activate the immune system and enhance the response against a specific pneumococcal antigen. At present, studies are being carried out in the nasopharynx to analyze the immune mechanisms induced at this level by the probiotic strain alone and associated with pneumococcal antigens. In previous studies, it was demonstrated that the oral administration of *L. casei *induced an increase in IgA cells in the bronchus-associated lymphoid tissue (BALT) in lung [44,45]. In contrast, CD4+ and CD8+ T lymphocytes were not able to migrate to sites distant from the intestine [9,44,45], although T-cell activation cannot be excluded as a cytokine source. According to these results, it seems likely that the direct stimulation at the nasopharynx level induced the IgA cells from the NALT, which would favour IgA production in the induction site and also in lung and serum by the "IgA cycle". The adjuvant effect of LcV and LcM over the specific humoral immune response was evident and it was coincident with the capacity of this lactobacillus to induce the Th2-cytokine IL-4 in NL, BAL and serum when it was nasally administered either alone (live and dead) or combined with PppA. In addition, the levels of specific antibodies induced by PppA+LcV and PppA+LcM immunization remained elevated, even 45 days after the last immunization, an essential condition for a vaccine. Nasal colonization is a crucial step in the progression of a pneumococcal infection and the high levels of specific antibodies induced by immunization with PppA+LcV and PppA+LcM in nasal and bronchoalveolar lavages would be involved in the reduction in nasopharynx and lung colonization by the pathogen. In this sense, IL-10 plays a key role in the modulation of the immune response induced after pneumococcal infection, limiting the inflammatory immune response and stimulating antibody production. Zhang *et al*. [46] demonstrated the regulatory role of IL10 in B-cell antibody responses against pneumococcal proteins. The authors suggest that IL-10 and INF-gamma are key regulators of mucosal anti-pneumococcal protein antibody production in the nasopharynx and are important in local protection against pneumococci. On the other hand, in a previous report, we showed that oral administration of *L. casei *beneficially regulates the balance between tumor necrosis factor-alpha and interleukin 10, allowing a more effective immune response against infection and modulating the inflammatory response, with less damage to the lung [20]. Thus, we considered that IL-10 induction by PppA+LcV and PppA+LcM would favor the humoral specific immune response and would regulate the inflammatory response after pneumococcal infection; low IL-10 levels would compromise regulation of the host defense response against infectious challenge. On the other hand, CD4+ Th cells play a central role in orchestrating the adaptive immune response and, although they are historically called helpers by their role in activating B, their functions are more extended. Thus, considering that the cellular immune response is very important in the defence against *S. pneumoniae*, stimulation of T helper cells was studied on the basis of the cytokines profile induced by immunization. Our results showed that PppA+LcV and PppA+LcM immunizations were able to stimulate INF-γ release in NL, BAL and serum and that LcV and LcM alone induced a similar effect although to a lesser degree. The INF-γ Th1 cytokine enhances chemokine expression and promotes pulmonary neutrophil recruitment into the infected lung, playing an important role in the neutrophil-mediated host protective responses against pneumococcal infection. IL-2 was increased in mucosa and serum by PppA+Lc and PppA+LcM immunizations during almost all the periods assayed and this effect would partly account for the high level of specific antibodies observed on day 73 after the first immunization. Although the factors influencing the differentiation of memory cells are incompletely understood, some investigations support a role of IL-2 in memory cell differentiation [47,48]. This cytokine would exert a strong influence on the proliferative capacity and maintenance of memory cells, which would be a desirable characteristic in the selection of an efficacious long-term vaccine. Moreover, IL-17 was increased following PppA+LcVand PppA+LcM vaccination in both the mucosal and systemic compartments. Recent studies have demonstrated that IL-17 plays a critical role in the recruitment of phagocytes that leads to the clearance of *S. pneumoniae *colonization from the mucosal surface of the nasopharynx. In addition, IL-17 would mediate the death of *S. pneumoniae *in the presence or absence of specific antibodies [49,50]. Thus, IL-17 induction by PppA+LcV and PppA+LcM immunization would play an important role in the reduction of colonization and increased survival of the vaccinated mice in the respiratory and intraperitoneal challenge assays. In contrast, PppA increased this cytokine only in NL and serum on some of the days assessed. Recent reports indicated that vaccine-induced IL-17 enhanced pathogen death by phagocytic cells through an increase in chemokines induction by epithelial cells that resulted in phagocytes recruitment and also by an increase in antimicrobial peptides implicated in the control of extracellular pathogens [51].

The induction and maintenance of antigen specific T-cell responses is an essential feature in the protection against *S. pneumoniae *that should be harnessed in the design of effective mucosal vaccines. The effectiveness of nasal immunization by combination of PppA with live and heat-killed *L. casei *was evident in challenge assays carried out on day 30, after the third immunization. Thus, PppA+LcV and PppA+LcM vaccination considerably reduced pathogen counts in NL and prevented lung and blood colonization when animals were challenged nasally. In addition, these treatments allowed the survival of 50-60% of the mice after peritoneal challenge with the pathogen. Immunization with PppA alone could fail to provide adequate protection against pneumococcal infection partly because it does not induce high specific antibody levels but mainly because it would not be able to stimulate effector and memory cells in an efficient way. In this sense, nasal immunization with PppA was not a good inducer of IL-2, IL-17 and INF-γ, which are important factors in the defence against *S. pneumoniae*. In contrast, vaccination with PppA+Lc (LcV and LcM) was able to induce a good specific humoral and cellular immune response in the mucosal and systemic compartments. Our results indicate that *L. casei *431 can act as a good mucosal adjuvant, allowing a potent specific immune response when it is associated with a pneumococcal antigen, and that the viability of the strain is not a crucial condition for stimulating the antigen-specific immune response. A recent report [52] demonstrated that nasal immunization of mice with a combination of PspA5 and whole cell *Bordetella pertussis *vaccine (wP) or wP with low levels of LPS (wP _low_) was able to protect against a respiratory lethal challenge with *S. pneumoniae*. However, the use of dead lactic acid bacteria (Gram+) as a nasal adjuvant represents a novel alternative for the development of mucosal vaccines using proteic antigens. This is the first report that considers the association of a specific pneumococcal antigen with a non-viable probiotic strain as an adjuvant for the development of a nasal vaccine. In addition, it is the first study dealing with the possible mechanisms involved in long-lasting specific systemic and mucosal immunity induced after nasal vaccination. The use of dead microorganisms would allow the safe application of potential vaccines in immunocompromised hosts and elderly people without the risks associated with the use of live bacteria [53]. The effect of live *Lactobacillus casei *431 as an adjuvant is not comparable with other studies [10] because "adjuvanticity" is characteristic of each strain and must be evaluated for a particular purpose. Thus, the design of a nasal vaccine based on pneumococcal antigens and heat-killed *L. casei *emerges as a safe and effective strategy for the prevention of pneumococcal infections and opens new possibilities for the application of dead lactic acid bacteria in the protection against other pathogens.

## Conclusions

In this work, we demonstrated that nasal immunization with live and heat-killed *Lactobacillus casei *associated to pneumococcal antigen was able to provide effective protection against pneumoccocal infection in mice. The results obtained in animal models do not guarantee their effectiveness in humans; however, they enable the establishment of scientific bases for subsequent testing in humans. In this sense, the use of vaccine formulations containing dead organisms is safer and more attractive for the design of human clinical trials in the short term.

## Methods

### Microorganisms and culture conditions

Recombinant *E. coli*-PppA was obtained in our laboratory and the development of this strain was described in a previous report from our work group [17]. Briefly, the *pppA *gene was amplified from the chromosomal DNA of *S. pneumoniae *T14 with EC1 and EC2 primers (EC1 (forward): **5'*****GGGG**CCATGGCTTGTAGAATTGAAAAAAGAA 3'*, EC2 (reverse): 5'*GGGGTCGACTAAACCAGGTGCTTGTCCAAGTTC 3'*). The PCR product was purified from agarose gel using home-made silica beads and treated with the restriction enzymes *Nco*I and *Sal*I. Then, the *pppA *gene was ligated into the *Nco*I and *Sal*I sites of pET28b using T4 DNA ligase (Promega). A recombinant plasmid containing the *pppA *gene, named pET-PppA, was first recovered from *E. coli *DH10B cells and then transformed into BL21 (DE3) cells for expression. BL21(DE3) (pET-PppA) cells were grown with aeration until they reached an OD_600 _of 0.8 in LB medium supplemented with neomycin (50 μg/ml) and induced with 1 mM IPTG (isopropyl-β-D-thiogalactopyranoside) (Sigma) for 2 h. Whole-cell lysates were prepared, cell debris was removed by centrifugation (20 min, 13,000 rpm, 4°C), and the proteins present in the supernatant were run on a 12% SDS-PAGE gel to confirm expression of the recombinant PppA (rPppA). rPppA was purified from BL21(DE3) (pET-PppA) as described below.

*Lactobacillus casei *CRL 431 (LcV) [20,21], obtained from the CERELA culture collection, was cultured for 8 h at 37°C (final log phase) in Man-Rogosa-Sharpe broth (Oxoid), harvested and washed with sterile 0.01 M phosphate buffer saline (PBS), pH 7.2. The bacterial suspension was adjusted to the desired concentration (10^9 ^cell/day/mouse) for its later administration by the nasal route. Heat-killed *L. casei *(LcM) was prepared by heating bacteria in a water bath at 80°C for 30 min and the lack of bacterial growth was confirmed using MRS agar plates.

*Streptococcus pneumoniae *serotype 14, kindly provided by Dr. M. Regueira from the Laboratory of Clinical Bacteriology, National Institute of Infectious Diseases, Argentina, was used. Freshly grown colonies of *S. pneumoniae *were suspended in THB and incubated at 37°C until the log phase was reached [17]. The pathogens were harvested by centrifugation at 3600 *g *for 10 min at 4°C and washed three times with sterile PBS. Then, the cell concentration of the pathogen was adjusted to the dose used in the respiratory (10^6 ^cells/mouse) and peritoneal (10^8 ^cells/mouse) challenge assays.

### Pneumococcal antigen

Recombinant PppA (rPppA) was purified using a His-Bind purification kit (Novagen) and visualized by electrophoresis on 12% SDS-polyacrylamide gels, as previously described [17]. The reagent and PppA solution were tested by the E-toxate test for LPS (Sigma) and shown to be below the limit of detection (2 pg/ml). rPppA concentration was determined by Bradford's method and 10 ug of this protein was used in immunization protocols.

### Mice

Three-week-old male and female Swiss albino mice were obtained from the closed colony at CERELA and each experimental group consisted of five or six mice for each period evaluated. During all experiments, animals were supplied with balanced rodent food and water *ad libitum*. Experiments were approved by the Animal Care and Ethics committee at CERELA.

### Nasal immunization protocol

Young mice were nasally immunized with 30 microlitres of a mixture of 5 ug of rPppA plus live (LcV) and heat-killed (LcM) *L. casei *(10^9 ^CFU) as the mucosal adjuvant. LcV and LCM suspension were prepared in PBS. The immunization was carried out using a protocol that comprised 3 successive administrations that included two consecutive days each time with a 14-day interval between them (days 0-1, 14-15 and 28-29). Groups that received LcV, LcM, rPppA and phosphate-buffered saline solution (PBS) were used as controls. Fourteen days after each immunization and also at days 58 and 73 after the first immunization, samples of nasal and bronchoalveolar lavages (NL, BAL) and serum were collected. For nasal lavage collection, a catheter attached to a syringe was inserted into the nostril and a total of 0.3 ml of PBS was injected. This procedure was carried out twice and the outflow was collected. Then, NL was centrifuged at 8,000 × *g *for 10 min to remove cell debris and clarified NL samples were collected. BAL samples were obtained according to the technique previously described [17,18]. Briefly, a catheter was inserted into the trachea of the mice and lungs were rinsed twice with 0.5 mL of sterile PBS. The fluid recovered from both rinses were pooled and after 10 min of centrifugation at 900 × *g *(at 4°C), the supernatants were collected. All fluids were stored at 70°C until analysis.

### Protection assays: respiratory and systemic challenges with S. pneumoniae

#### Respiratory infection

Experimental respiratory infection was induced as previouslydescribed. Thirty days after the 3^rd ^immunization, control and immunized mice were challenged intranasally with the pathogen by dripping 25 μl of an inoculum containing 10^6^CFU (log phase) in PBS into each nostril and allowing it to be inhaled. Mice were killed at 3 days after challenge with *S. pneumoniae *and their lungs were excised, weighed and homogenized in 5 ml sterile peptone water. Also, nasal lavages were collected as described above. Homogenates and nasal lavages were diluted appropriately, plated in duplicate on Columbia sheep blood agar and incubated for 18 h at 37°C; then, the number of colonies was counted. *S. pneumoniae *was identified by standard techniques [17,20] and the results were expressed as log of CFU/g of organ for lung and CFU/ml for nasal lavages. In addition, progression of bacterial growth to the bloodstream was monitored by sampling blood obtained through cardiac puncture with a heparinized syringe and plating on blood agar. Bacteremia was reported as CFU/ml.

#### Systemic infection

Intraperitoneal challenge experiments were carried out 30 days after the third immunization of the animals. *S. pneumoniae *serotype 14 was grown until cells reached their exponential phase under the conditions described above, and each mouse was infected intraperitoneally with 100 ul of PBS containing 10^8 ^cells of the pathogen. Each experimental group consisted of 14 mice and survival was closely monitored for 21 days.

### Enzyme-linked immunosorbent assay (ELISA) for anti-PppA antibodies

Serum and BAL antibodies against PppA protein were determined by ELISA, according to previous experiments [17,37]. Briefly, plates were coated with rPppA (100 ul of a 5 ug/ml stock in sodium carbonate-bicarbonate buffer, pH 9.6, per well). Non-specific protein binding sites were blocked with PBS containing 5% non-fat milk. Samples were diluted (Serum 1:100; BAL 1:20 and NL 1:4) with PBS containing 0.05% (v/v) Tween 20 (PBS-T). Peroxidase-conjugated goat anti-mouse IgA and IgG (Fc specific; Sigma Chemical, St Louis, MO, USA) were diluted (1:500) in PBS-T. Antibodies were revealed with a substrate solution [o-phenylenediamine (Sigma Chemical)] in citrate-phosphate buffer (pH 5, containing 0.05% H_2_O_2_) and the reaction was stopped by the addition of H_2_SO_4 _1 M. Readings were carried out at 493 nm (VERSAmax Tunable microplate reader; MDS Analytical Technologies, Sunnyvale, CA, USA) and samples were considered negative for the presence of specific antibodies when OD493 < 0.1.

### Cytokine concentration

Cytokine concentrations in NL, BAL and serum were measured by mouse Th1/Th2 ELISA Ready SET Go! Kit (BD Bioscience, San Diego, CA, USA), including interleukin (IL)-2 and interferon (IFN)-γ as Th1-type, IL-4 and IL-10 as Th2-type cytokines. The IL-17A as a Th17-type cytokine was also measured using the ELISA kit from e-Bioscience (BD Biosciences). The sensitivity of assays for each cytokine was as follows: 4 pg/ml for IL-2, IFN-γ and tumour necrosis factor (TNF)-α, and 2 pg/ml for IL -4 and IL-10 and IL-17 4 pg/ml.

### Statistical Analyses

Experiments were performed in triplicate and results were expressed as mean ± standard deviation (SD). After verification of a normal distribution of data, significant differences between means were determined by analysis of variance (ANOVA) with Fisher's least significant difference (LSD) *post hoc *test using the StatGraphics software (Manugistics, Rockville, MD, USA). Differences were considered significant at *P *< 0.05.

## Abbreviations

NL: nasal lavages; BAL: Bronchoalveolar lavages, heat killed *Lactobacillus casei: *LcM, live *Lactobacillus casei: *LcV, pneumococcal protective protein A: PppA.

## Authors' contributions

EV and MM contributed in designing the study and performed the experimental assays and analysis of results. MM performed the statistical analysis, prepared and wrote the manuscript. All authors read and approved the final manuscript.
